# Supplementary material for: Chromosome-Level Assembly of Flowering Cherry (Prunus campanulata) Provides Insight into Anthocyanin Accumulation
Source: Genes (Basel). 2023 Feb 2;14(2):389. doi: 10.3390/genes14020389 (PMC9957189; doi:10.3390/genes14020389)
Supplement: Supplementary file 1 [file genes-14-00389-s001.zip › Figure/Figure caption.pdf]

Figure S1. Estimation of the genome size of *P. campanulata*, based on *k*-mer analysis (k = 17). The x-axis is *k*-mer depth; the y-axis is the proportion that represents the frequency at that depth divided by the total frequency of all depths.

Figure S2. GC content analysis of *P. campanulata* genome based on Illumina reads.

Figure S3. Divergence analyses of transposable elements in the genome of *P. campanulata* based on Kimura distance (K-value from 0 to 60).

Figure S4. Synteny analysis between *P. campanulata*, *P. avium*, and *P. persica* genomes. (A) Collinearity pattern of gene pairs between *P. campanulata* and *P. avium*. (B) Collinearity pattern of gene pairs between *P. campanulata* and *P. persica*.

Figure S5. Genomic synteny between *P. campanulata* and *P*. × *kanzakura* "Kawazu-zakura". (A) Collinearity pattern of genomes between *P. campanulata* and *C. campanulata* haplotype. (B) Collinearity pattern of genomes between *P. campanulata* and *C. spachiana* haplotype. Pca: *P. campanulata*; Cam: *C. campanulata* haplotype; Spe: *C. spachiana* haplotype.
