# Supplementary material for: Chromosome-Level Assembly of Flowering Cherry (Prunus campanulata) Provides Insight into Anthocyanin Accumulation
Source: Genes (Basel). 2023 Feb 2;14(2):389. doi: 10.3390/genes14020389 (PMC9957189; doi:10.3390/genes14020389)
Supplement: Supplementary file 1 [file genes-14-00389-s001.zip › Table/TableS1-S13.pdf]

Table S1. Summary of sequencing data of *P. campanulata*.

Method	Sequencing plantform	Library size	Total data (Gb)	Reads number	Sequence coverage (X)
Pair-end sequencing	Illumina HiSeqXTen	350 bp	53.47	105,323,237	163.07
PacBio SMRT	Pacbio Sequel	20 Kb	42.73	5,198,616	130.32
10X Genomics Chromium	Illumina HiSeqXTen	350 bp	39.08	130,279,189	119.19
Hi-C	Illumina HiSeqXTen	350 bp	42.20	118,522,234	129.05

Table S2. Summary of Hi-C assembly.

	Input assembly	LACHESIS assembly
Total Length	300.48 Mb	300.08 Mb
L50/N50	26 Scaffolds; 3.29 Mb	4 Scaffolds; 32.53 Mb
L90/N90	102 Scaffolds; 0.68 Mb	8 Scaffolds; 26.95 Mb
Longest Scaffolds	12.08 Mb	51.49 Mb
Number of Scaffolds	492	329

Table S3. Chromosome clusters using Hi-C sequencing data.

Group	Number of scaffolds	Length (bp)
Pca1	38	51,585,711
Pca2	74	39,022,630
Pca3	60	37,392,320
Pca4	58	33,163,670
Pca5	69	32,973,666
Pca6	50	31,784,716
Pca7	35	30,424,867
Pca8	44	27,274,175
Total	428	283,621,755

Table S4. Quality assessment of the assembled genome of *P. campanulata* using BUSCO and CEGMA.

Type	BUSCO		CEGMA	
	Proteins	Percentage (%)	Proteins	Percentage (%)
Complete (BUSCO and CEGMA)	1,390	96.5	235	94.8
Complete and single-copy (BUSCO)	1,289	89.5		

Complete and duplicated (BUSCO)	101	7.0		
Fragmented (BUSCO)	16	1.1		
Complete and partial (CEGMA)			240	96.8
Missing (BUSCO)	34	2.4		

Table S5. Summary of function annotation of protein-coding genes in *P. campanulata* using different databases.

**Database**		**Annotated Number**	**Annotated Percentage (%)**
NR		26,322	93.00
Swiss-Prot		20,711	73.20
KEGG		20,006	70.70
	All	20,587	72.80
InterPro	Pfam	19,260	68.10
	GO	14,215	50.20
Annotated		26,352	93.10
Total		28,291	-

Table S6. Summary of non-coding RNAs annotation in *P. campanulata*.

**Type**		**Copy(w*)**	**Average length(bp)**	**Total length(bp)**	**Percentage of genome (%)**
miRNA		562	136.49	76,710	2.6
tRNA		721	74.89	53,994	1.8
rRNA	rRNA	330	319.58	105,463	3.5
	18S	61	1132.93	69,109	2.3
	28S	152	140.05	21,288	0.7
	5.8S	42	146.07	6,135	0.2
	5S	75	119.08	8,931	0.3
snRNA	snRNA	617	111.42	68,856	2.3
	CD-box	428	102.89	44,039	1.5
	HACA-box	40	121.62	4,865	0.2
	splicing	149	133.04	19,823	0.7

Table S7. The numbers of orthologs among 11 species.

**Species**	**Single-copy orthologs**	**Multiple-copy orthologs**	**Unique**	**Other orthologs**

P. campanulata	5658	6240	500	10121
P. persica	5908	6297	1523	10859
P. mume	5966	5935	117	9496
P. avim	6071	5404	5819	13715
Pyrus bretschneideri	3129	15496	747	12035
Malus domestica	2581	16372	1110	12818
Fragaria vesca	6032	5622	1659	9359
Potentilla micrantha	6104	5197	2872	10420
Rosa chinensis	5780	7302	1300	12630
Rubus occidentalis	6009	5584	3650	9632
Arabidopsis thaliana	5019	8285	3230	6408

Table S8. The numbers of collinear gene pairs in the chromosomes of *P. campanulata*, *P. avium*, and *P. persica*.

**Chromosome**	***P. campanulata* vs *P. avium***		***P. campanulata* vs *P. persica***	
	P. campanulata	***P. avium***	***P. campanulata***	***P. persica***
Chr1	3538	3388	4118	4046
Chr2	1731	1752	2152	1970
Chr3	1984	1662	2551	1944
Chr4	1751	1707	2137	1977
Chr5	1605	1439	1938	1761
Chr6	1597	1924	1890	2443
Chr7	1476	1575	1812	1921
Chr8	1557	1452	1785	1752
Scaffolds	90	115	163	186

Table S9. Syntenic regions between *P. campanulata* and *C. campanulata* haplotype of *P.* × *kanzakura* "Kawazu-zakura".

Table S10. Syntenic regions between *P. campanulata* and *C. spachiana* haplotype of *P.* × *kanzakura* "Kawazu-zakura".

Table S11. Significantly enriched GO terms for family genes under positive selection.

**GO term**	**GO class**	**Adjusted *P*-value**	**Gene number**
nucleic acid metabolic process	BP	0.020770848	75
cellular aromatic compound metabolic process	BP	0.023149632	88

heterocycle metabolic process	BP	0.023149632	88
organic cyclic compound metabolic process	BP	0.023149632	89
nucleobase-containing compound metabolic process	BP	0.023149632	83
phagocytosis	BP	0.023149632	3
cellular nitrogen compound metabolic process	BP	0.023149632	88
bile acid:sodium symporter activity	MF	0.0288106	3
ncRNA processing	BP	0.03893984	9
RNA processing	BP	0.0456541	16
RNA metabolic process	BP	0.049872408	59

Table S12. Significantly enriched GO terms for family genes under expansion and contraction.

**GO term**	**GO class**	**Adjusted *P*-value**	**Gene number**	**Type**
phosphoglycerate kinase activity	MF	2.99E-07	4	Expansion
terpene synthase activity	MF	2.39E-05	4	Expansion
translation release factor activity, codon specific	MF	2.39E-05	4	Expansion
sequence-specific DNA binding	MF	3.39E-05	11	Expansion
translational termination	BP	4.31E-05	4	Expansion
generation of precursor metabolites and energy	BP	0.000213184	6	Expansion
sequence-specific DNA binding transcription factor activity	MF	0.000779133	11	Expansion
glycolytic process	BP	0.001980865	4	Expansion
oxidoreductase activity	MF	0.003235302	6	Expansion
ATP synthesis coupled electron transport	BP	0.004563201	2	Expansion
adenyl ribonucleotide binding	MF	8.01E-86	136	Contraction
purine ribonucleoside binding	MF	7.01E-72	136	Contraction
nucleotide binding	MF	1.33E-64	140	Contraction
anion binding	MF	2.59E-63	140	Contraction
ADP binding	MF	3.23E-46	43	Contraction
protein tyrosine kinase activity	MF	4.72E-45	63	Contraction
protein phosphorylation	BP	4.92E-43	67	Contraction
protein kinase activity	MF	2.56E-42	67	Contraction
ion binding	MF	1.26E-36	166	Contraction

defense response	BP	1.18E-35	41	Contraction

Table S13. Significantly enriched KEGG terms for family genes under expansion and contraction.

**Map title**	**Adjusted *P*-value**	**Gene number**	**Type**
Ribosome biogenesis in eukaryotes	0.005609269	6	Expansion
Diterpenoid biosynthesis	0.006402294	4	Expansion
Flavonoid biosynthesis	0.008391851	5	Expansion
ABC transporters	0.023259336	4	Expansion
Circadian rhythm - plant	0.026329428	4	Expansion
Carbon fixation in photosynthetic organisms	0.029486305	4	Expansion
Ribosome	0.046266724	7	Expansion
Cyanoamino acid metabolism	2.61E-30	38	Contraction
Phenylpropanoid biosynthesis	1.02E-12	25	Contraction
ABC transporters	4.45E-12	21	Contraction
Plant-pathogen interaction	1.38E-11	35	Contraction
Tropane, piperidine and pyridine alkaloid biosynthesis	2.76E-06	10	Contraction
Isoquinoline alkaloid biosynthesis	1.71E-05	10	Contraction
Phenylalanine metabolism	3.83E-05	10	Contraction
beta-Alanine metabolism	7.56E-05	10	Contraction
Stilbenoid, diarylheptanoid and gingerol biosynthesis	7.56E-05	11	Contraction
Tyrosine metabolism	0.00023254	10	Contraction
Glycine, serine and threonine metabolism	0.001362489	10	Contraction

Table S14. The gene ID of MYB genes identified in the genome of *P. campanulata.*

Table S15. The FPKM values of the *PcMYB* genes in the various tissues of *P. campanulata.*

Table S16. Summary of TE contents in *P. campanulata*, *P. avium* “Tieton”, *P. avium* “Satonishiki”, *C. serrulata*, and *P. fruticosa*.
