# Supplementary figures and images for: Chromosome-Level Assembly of Flowering Cherry (Prunus campanulata) Provides Insight into Anthocyanin Accumulation
Source: Genes (Basel). 2023 Feb 2;14(2):389. doi: 10.3390/genes14020389 (PMC9957189; doi:10.3390/genes14020389)

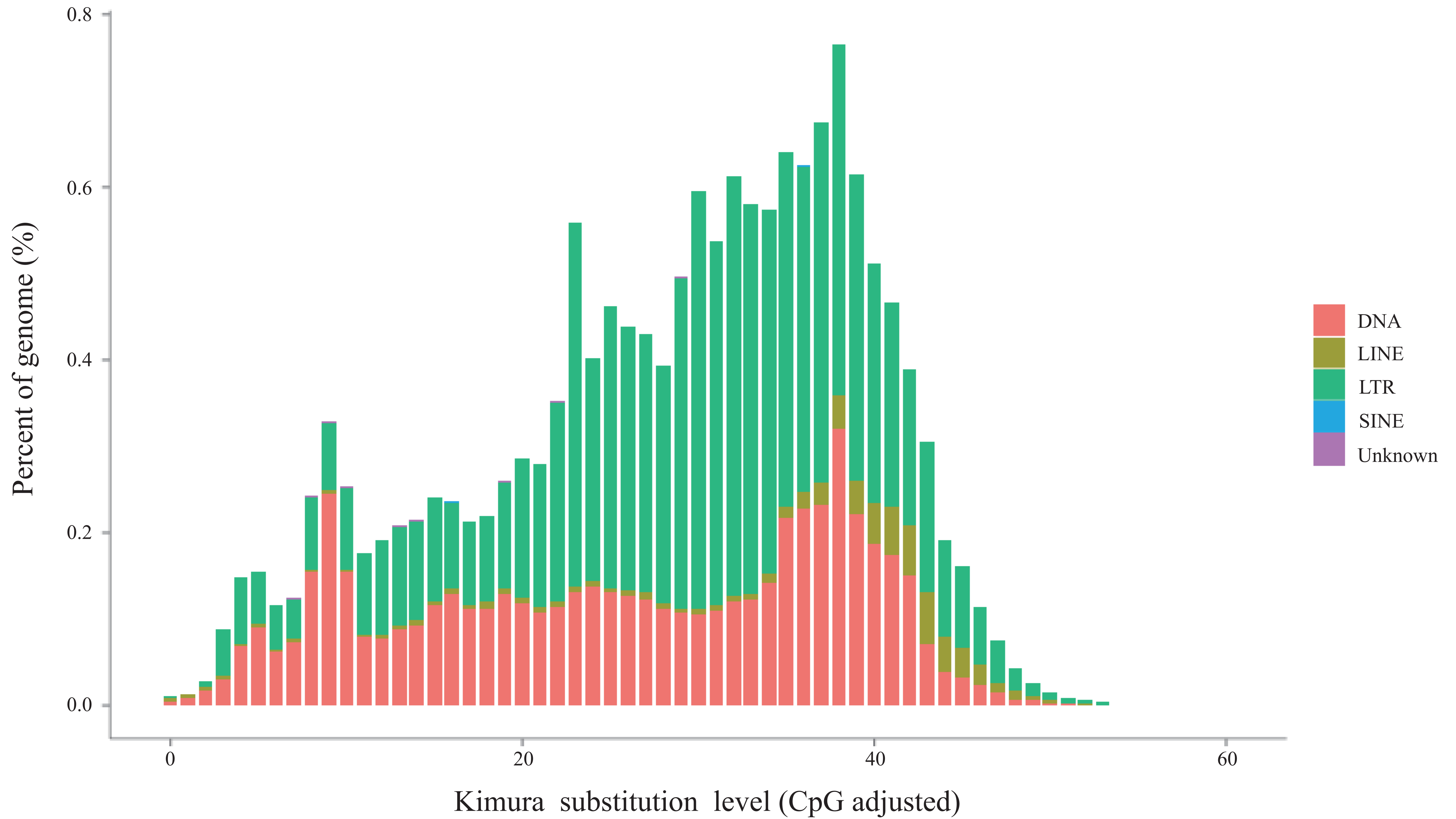

Supplement: Supplementary file 1 [file genes-14-00389-s001.zip › Figure/Fig S3 TEs divergence.pdf]

A

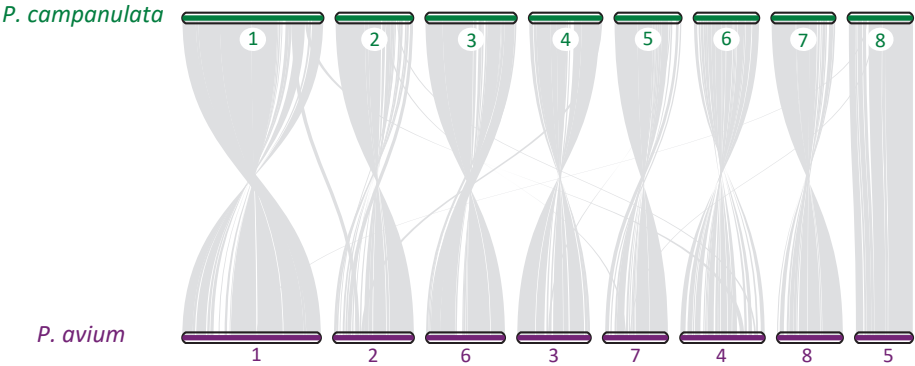

B

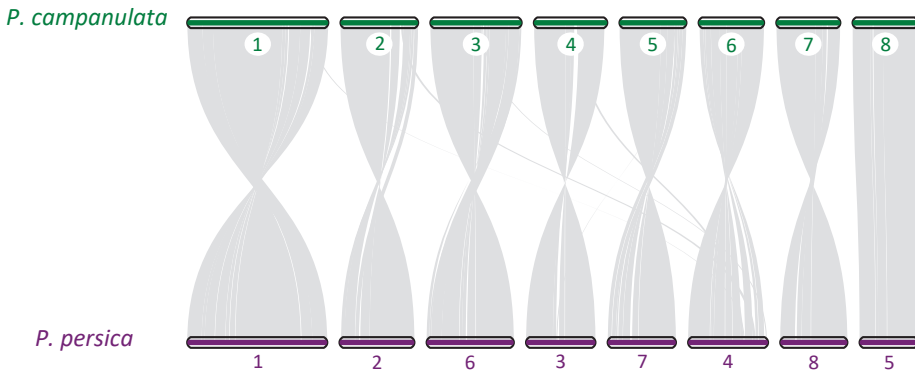

Supplement: Supplementary file 1 [file genes-14-00389-s001.zip › Figure/Fig S4 Gene synteny.pdf]

A

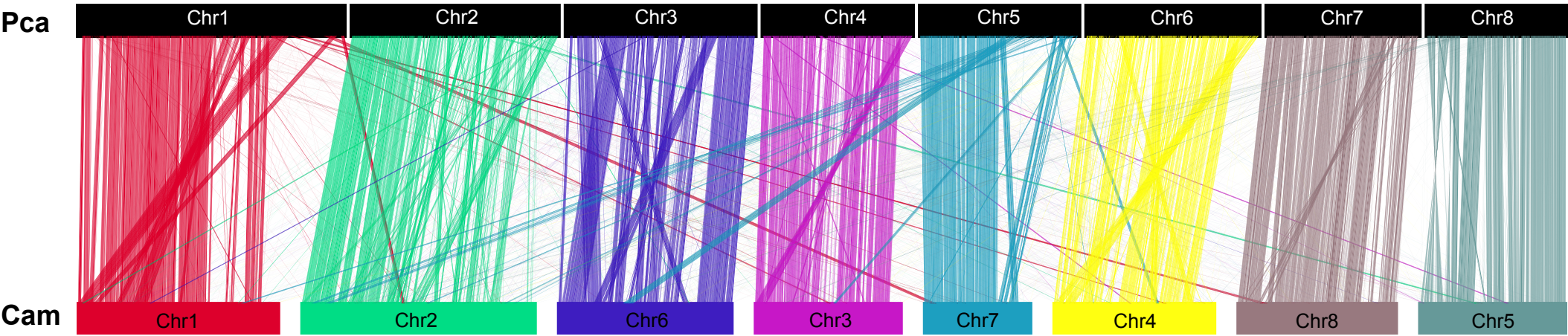

B

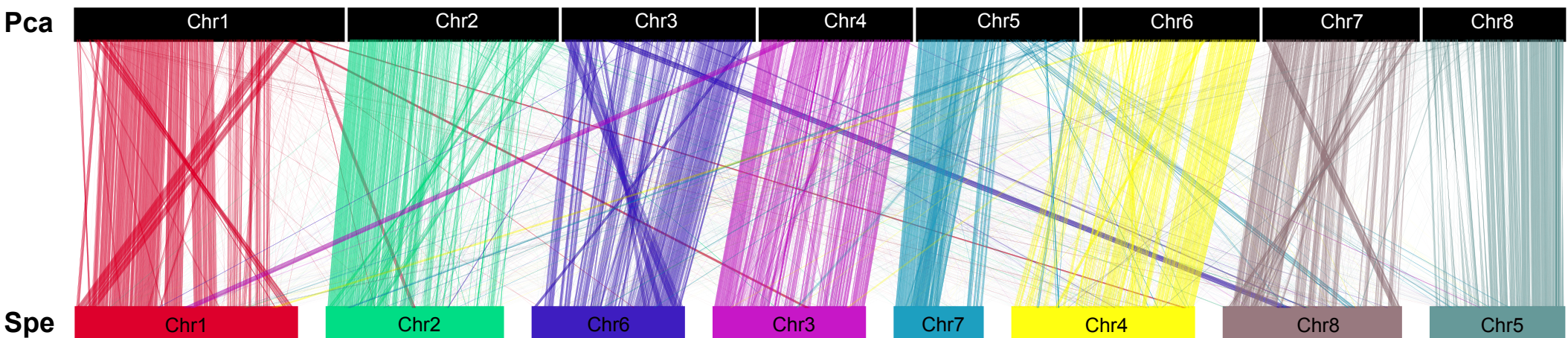

Supplement: Supplementary file 1 [file genes-14-00389-s001.zip › Figure/Fig S5 Genomic synteny.pdf]

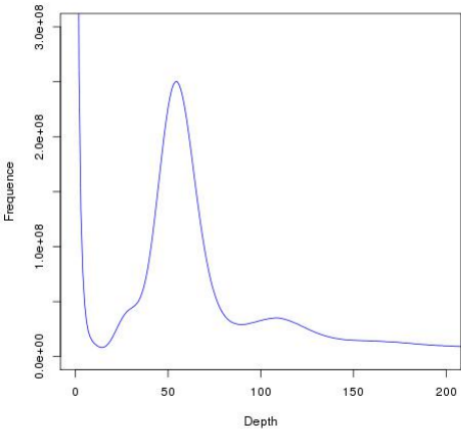

Supplement: Supplementary file 1 [file genes-14-00389-s001.zip › Figure/FigS1 Estimation of genome size.pdf]

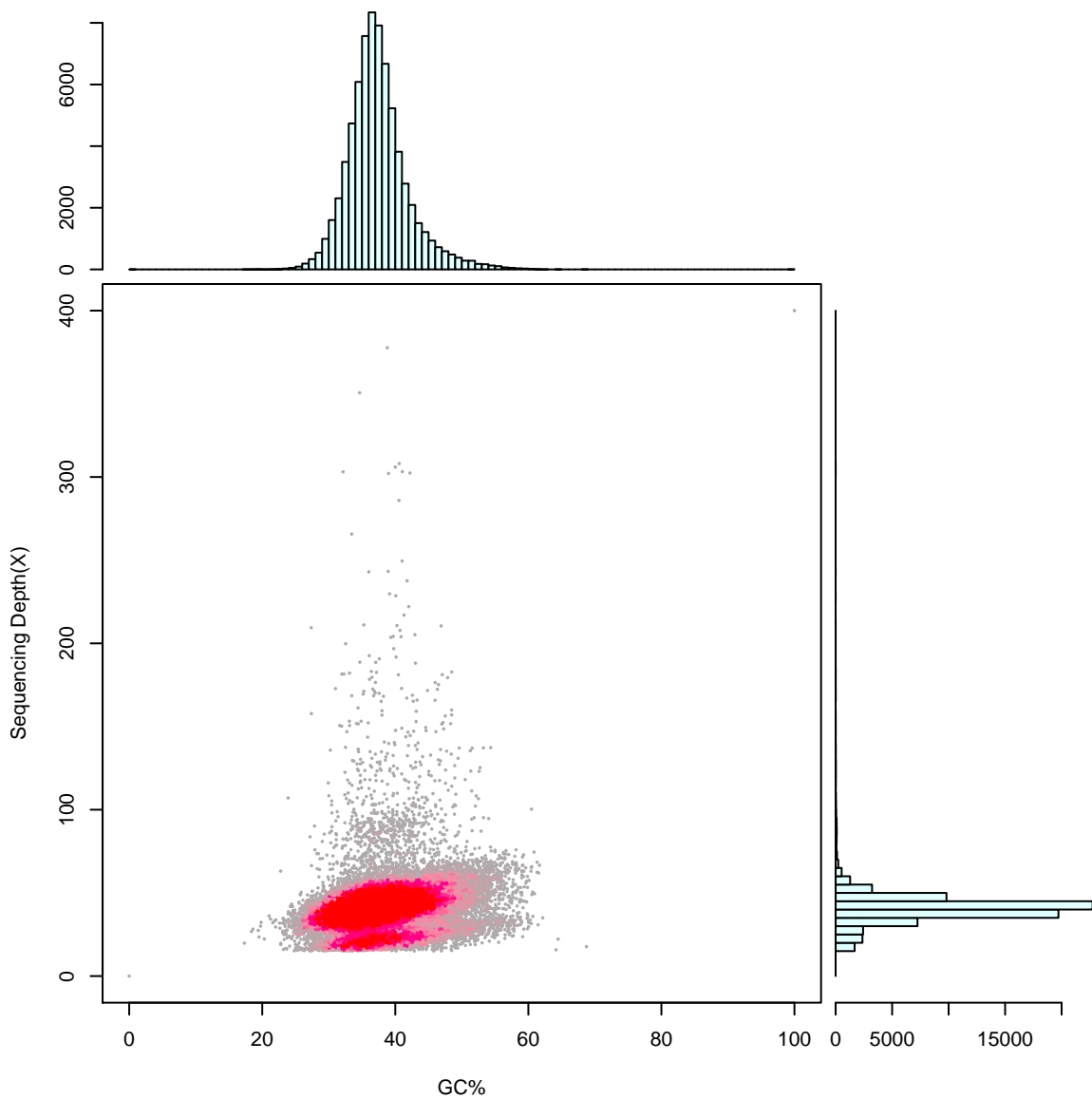

Supplement: Supplementary file 1 [file genes-14-00389-s001.zip › Figure/FigS2 GC content.pdf]
